# Cluster analysis exploring the impact of childhood neglect on cognitive function in patients with bipolar disorder

**DOI:** 10.1186/s40345-024-00335-w

**Published:** 2024-04-27

**Authors:** Yuan-Zhi Hsueh, Cho-Yin Huang, Po-Hsiu Kuo, Ying-Chih Cheng, Ming-Chyi Huang, Chih Chiang Chiu, Chian-Jue Kuo, Po-Yu Chen, Wen-Yin Chen

**Affiliations:** 1 Department of Psychiatry, Taipei City Psychiatric Center, Taipei City Hospital, Songde branch, Taipei, Taiwan; 2 Institute of Health Behaviors and Community Sciences, College of Public Health, National Taiwan University, Taipei, Taiwan; 3 Department of Psychiatry, School of Medicine, College of Medicine, Taipei Medical University, Taipei, Taiwan; 4 Department of Psychiatry, China Medical University Hsinchu Hospital, China Medical University, Hsinchu, Taiwan; 5 Department of Public Health, College of Public Health, National Taiwan University, Taipei, Taiwan; 6 Department of Psychiatry, National Taiwan University Hospital, Taipei, Taiwan; 7 School of Medicine, College of Medicine, Fu Jen Catholic University, New Taipei, Taiwan; 8 Psychiatric Research Center, Taipei Medical University Hospital, Taipei, Taiwan; 9 Institute of Epidemiology and Preventive Medicine, College of Public Health, National Taiwan University, Taipei, Taiwan

**Keywords:** Bipolar disorder, Childhood trauma questionnaire, Neglect, Cluster analysis, Working memory

## Abstract

**Background:**

Bipolar disorder (BD) is a severe mental disorder related to neurocognitive deficits. Exposure to childhood trauma is associated with worse cognitive performance. Different compositions of childhood trauma in BD and their impacts on cognition are rarely reported.

**Methods:**

We used the Brief Assessment of Cognition in Affective Disorders (BAC-A) to assess cognitive performance and the Chinese version of the Short Form of the Childhood Trauma Questionnaire (C-CTQ-SF) to assess childhood trauma experience among 55 euthymic BD patients. Cluster analysis was applied to dissect their childhood trauma experiences, which revealed three distinct clusters: a low trauma group, neglect-focus group, and multiple-trauma-experience group. We compared the cognitive function between the three clusters and used a generalized linear model to evaluate the impact of childhood neglect on cognitive domains.

**Results:**

The neglect-focus cluster showed prominent exposures to physical and emotional neglect (41.8%). BD patients in this cluster performed worse in BAC-A compared with patients in the multiple trauma cluster, especially in working memory and processing speed. The neglect-focus group revealed a significant negative effect on the composite score (ß = -0.904, *p* = 0.025) and working memory (ß = -1.150, *p* = 0.002) after adjusting sex, age, education year, BMI and total psychotropic defined daily dose.

**Conclusions:**

Distinct patterns of childhood trauma experience are seen in BD patients and are related with different cognitive profiles. Early exposure of neglect-focus trauma was associated with the worst cognitive performance in current study. Further studies investigating the intensity of the neglect, as well as individual resilience and coping mechanisms in BD, are warranted.

**Supplementary Information:**

The online version contains supplementary material available at 10.1186/s40345-024-00335-w.

## Introduction

Bipolar disorder (BD) is a chronic mental illness with features of fluctuations in mood state and has a lifetime prevalence ranging between 0.1 and 2.4% (Grande et al. 2016; Rowland and Marwaha 2018). It is also one of the most disabling conditions, causing high days out of role and disability-adjusted life years (Alonso et al. 2011; He et al. 2020). Among patients, impairment crosses neurocognitive domains, including attention, verbal learning and memory, and executive functions had been noted (Solé et al. 2017). Although there is no specific intervention with pro-cognitive effects so far, multiple factors such as age, education level, illness duration, and clinical course are associated with the cognitive impairment (Mann-Wrobel et al. 2011; Tamura et al. 2021). Adverse childhood experiences are not uncommon in BD patients, and studies have revealed an association between childhood adversities and poor cognitive presentation among individuals (Poletti et al. 2014; Rokita et al. 2018). A previous study targeting major depression and BD patients also highlighted that cognitive impairment is especially observed in subjects exposed to great childhood adversities, indicating the importance of early experience in the cognitive functions in mood disorders (Poletti et al. 2017).

Exposure to childhood adversity has been noticed as a risk factor of poor health outcomes, including both physical and mental health (Hustedde 2021; Petruccelli et al. 2019; Sonu et al. 2019). Studies also suggested that childhood trauma experience and its impact on outcomes may differ between genders (Xiao et al. 2020; Yue et al. 2023; Zhao and Wu 2022). Compared to general populations, BD patients usually have higher prevalence of childhood adversities, such as abuse, neglect, parenting absence, or familial economic difficulties (Bruni et al. 2018; Miskowiak et al. 2023). Childhood maltreatment is related to many unfavorable clinical courses in BD, including earlier onset age, more severe symptoms, and more risk of co-morbidities (Agnew-Blais and Danese 2016; Carbone et al. 2019; Caruso et al. 2021; Farias et al. 2019; Park et al. 2020; Sun et al. 2022). In addition, studies show that this population presents with increased emotional hyper-reactivity, more impulsivity, and more fear of negative evaluation (Janiri et al. 2020; Lucero et al. 2022; Richard-Lepouriel et al. 2019). All the negative impacts are harmful to occupational and executive function and may persist both during the active illness phase and in remission from BD (Cotter and Yung 2018; Hjelseng et al. 2022; Lund et al. 2020, 2022).

The latest systematic review suggested that childhood trauma subtypes may differentially influence specific cognitive abilities (Rosa et al. 2023). One study analyzing sub-categories of childhood trauma also showed that working memory impairments are related particularly to physical and emotional abuse in childhood, while psychosocial difficulties are related to physical and emotional neglect (Miskowiak et al. 2023). However, studies also mention that current binary categories or score approaches of childhood adversities might oversimplify their impact, and other empirically driven approaches may be warranted (Lacey and Minnis 2020). Some studies have shown that different clusters of childhood trauma experience are associated with different psychosocial outcomes in adults (Barboza 2018; Begemann et al. 2022; Zietz et al. 2020; Zuo et al. 2021). With cluster analysis, we can find association patterns between forms of child maltreatment (Matsumoto et al. 2021) and elucidate the mechanisms. It is important to consider the effects of co-occurrence of different types of adverse childhood experiences at the same time. However, no study has used cluster analysis to dissect the childhood trauma experience in BD and to reveal its impact on cognitive functions so far. We hypothesized that specific patterns of childhood trauma may exist in BD patients. Furthermore, these different patterns of childhood trauma may have varying impacts on their cognitive function. The aim of the current study is to conduct a trauma-driven cluster analysis to evaluate the differences of characterization and cognitive function between different childhood trauma experience clusters in BD.

## Method

### Participants

In this study, 55 individuals who had been diagnosed of BD type I according to the DSM-5 (Diagnostic and Statistical Manual of Mental Disorders, Fifth Edition) by licensed psychiatrists, were enrolled from the outpatient department of TCPC (Taipei City Psychiatric Center), a tertiary psychiatric hospital. All participants were more than 20 years old and were both euthymic and under stable medications (no change in medications in the previous 3 months).

The following exclusion criteria were applied: (1) diagnosis of substance use disorder (past or currently), with the exception of a nicotine use disorder; (2) medical condition associated with neurological symptoms or complications, such as brain injury or stroke; (3) pregnancy or active physical illness, such as such as renal impairment or hepatic failure; (4) diagnosis of intellectual disability, schizophrenia, or schizoaffective disorders; and (5) inability to complete the assessment or provide informed consent. Information regarding psychiatric comorbidities and the exclusion criteria was obtained through the Chinese version of the modified Schedule of Affective Disorder and Schizophrenia – Lifetime (SADS) and patients’ medical records. SADS is a collection of psychiatric diagnostic criteria and symptom rating scales organized as a semi-structured diagnostic interview (Hesselbrock et al. 1982). The study was approved by the Research Ethics Committee of Taipei City Hospital (TCHIRB-11,101,011). Written informed consent was obtained from all patients.

### Measurements

Demographic data and patients’ clinical characteristics were investigated though medical records and interviews by psychiatrists. Clinical data collected included the number of episodes (total, manic, mood episodes with psychotic features), age of disease onset, and duration of illness. Defined daily dose (DDD) was calculated according to the psychopharmacological medications that the patients used at the time of assessment, representing the assumed average maintenance dose per day for a drug used for its main indication in adults. The premorbid intelligence quotient (IQ) was estimated through the Adult Reading Test from the Wechsler Adult Intelligence Scale by a licensed psychologist. In this study, clinician-administered measures of the 17-item Hamilton Depression Rating Scale (HDRS) and the Young Mania Rating Scale (YMRS) were used to evaluate mood symptoms. Scores of both HDRS and YMRS ≤ 8 were defined as euthymia in this study (Sussman et al. 2007; Zimmerman et al. 2013).

Patients enrolled in this study were assessed with the Brief Assessment of Cognition in Affective Disorders (BAC-A). BAC-A has been used widely as a quick and reliable tool to assess cognitive performance in patients with a wide range of clinical affective disorders (Bauer et al. 2015; Chen et al. 2019). Usually, BAC-A was administered in around 35–60 min. Within this assessment, six neurocognitive domains were measured: verbal memory (list learning), motor speed (token motor task), working memory (digit sequencing task), verbal fluency (category instances and controlled oral word association test), processing speed (symbol coding), and executive function (tower of London) (Keefe et al. 2014). The score was then standardized to a Z score from a norm reference (Lee et al. 2018). Each subtest assessing cognitive impairment has demonstrated criterion validity and construct validity. In addition, the use in different cultures and language groups has also been validated (Wang et al. 2017).

The Short Form of the Childhood Trauma Questionnaire (CTQ-SF) was used to assess traumatic childhood experiences (Bernstein et al. 1997, 2003). The CTQ-SF is a 28-item retrospective self-report questionnaire evaluating multiple trauma subtypes, including physical abuse (PA), emotional abuse (EA), sexual abuse (SA), emotional neglect (EN), and physical neglect (PN). Three items were used for validity evaluation, and five items were used for each of the five types of maltreatment. A 5-point Likert-type response was used for each item to assess the frequency of trauma experience (1 = never, 2 = occasionally; 3 = sometimes; 4 = frequently; 5 = always). Each subscale score ranged from 5 to 25. The cut-off score for a “mild to moderate” degree of exposure for each subtype was 8 for physical abuse, 9 for emotional abuse, 6 for sexual abuse, 10 for emotional neglect, and 8 for physical neglect (Bernstein and Fink 1998; Häuser et al. 2011). The Chinese version of CTQ-SF (C-CTQ-SF) shows adequate reliability and validity in Chinese populations (Ying-Chih Cheng 2018).

### Statistical analyses

All data were analyzed using the software SPSS version 20 (SPSS Inc.). First, we compared the demographic and clinical characteristics, CTQ score, and number of childhood trauma experiences between genders among the BD patients, using Student’s t-tests to assess continuous variables. Normality of data was assessed using the Kolmogorov-Smirnov test. For non-normally distributed data, we used non-parametric Wilcoxon rank sum test for analysis. Second, a two-step cluster analysis was chosen, and clusters were identified based on childhood trauma experiences (PA, EA, SA, EN, and PN). For the purpose of identifying subgroups with more than two clusters (high-trauma and low-trauma), the number of clusters was set to be more than two. For the distance measure, the log-likelihood criterion was used. Both Schwarz’s Bayesian criterion (Karlsson Linnér et al. 2019) and the silhouette coefficient were used to compare cluster solutions. The silhouette coefficient was classified as poor (< 0.2), fair (0.2–0.5), or good solution quality (> 0.5). Fair or higher was considered acceptable clustering (Carbone et al. 2019). In the current dataset, the three-cluster solution had the lowest BIC value (231.302) and a silhouette coefficient of 0.4.

Demographic data including gender, clinical characteristics, and cognitive function between the three clusters were analyzed using ANOVA, and post-hoc comparisons were done if significant differences were noticed. Series of generalized linear models (GLMs) were performed to examine the effect of clusters on cognitive domains with the low trauma cluster as a reference after adjustment for confounding factors. The level of statistical significance was set at *p* < 0.05, two-tailed.

## Results

### Characteristics of participants among the clusters

Demographic data of the enrolled BD patients are presented in Table 1. We enrolled 55 patients (25 men and 30 women) with BD who were in stable mood condition. In the male group, the mean age was 49.08 years, and in the female group, the mean age was 48.27 years. There was no group difference between genders in demographic data and clinical characteristics except that females had a greater number of major depressive episodes compared with males.

**Table 1:** Demographic data among the enrolled bipolar patients

	Male (*N* = 25)	Female (*N* = 30)	*P*-value
Age	49.08(12.35)	48.27(11.31)	0.800
Estimated premorbid IQ	98.33(15.30)	99.50(14.29)	0.856
Disease onset age	26.80(11.03)	26.43(9.78)	0.897
Educational years	12.88(3.56)	13.80(2.70)	0.282
Number of total episodes	8.44(8.16)	8.86(4.78)	0.814
Number of manic episodes	6.44(7.50)	5.10(3.48)	0.394
Number of major depressive episodes	1.84 (2.09)	3.48 (3.30)	0.032*
Number of psychotic mood episodes	4.44(7.69)	2.70(3.38)	0.269
Total score of CTQ	57.44(12.26)	53.93(10.16)	0.251
Number of CTQ	2.96(1.43)	2.87(1.70)	0.828

CTQ, Childhood Trauma Questionnaire; IQ, Intelligence Quotient

*p < 0.05

**Fig. 1:**
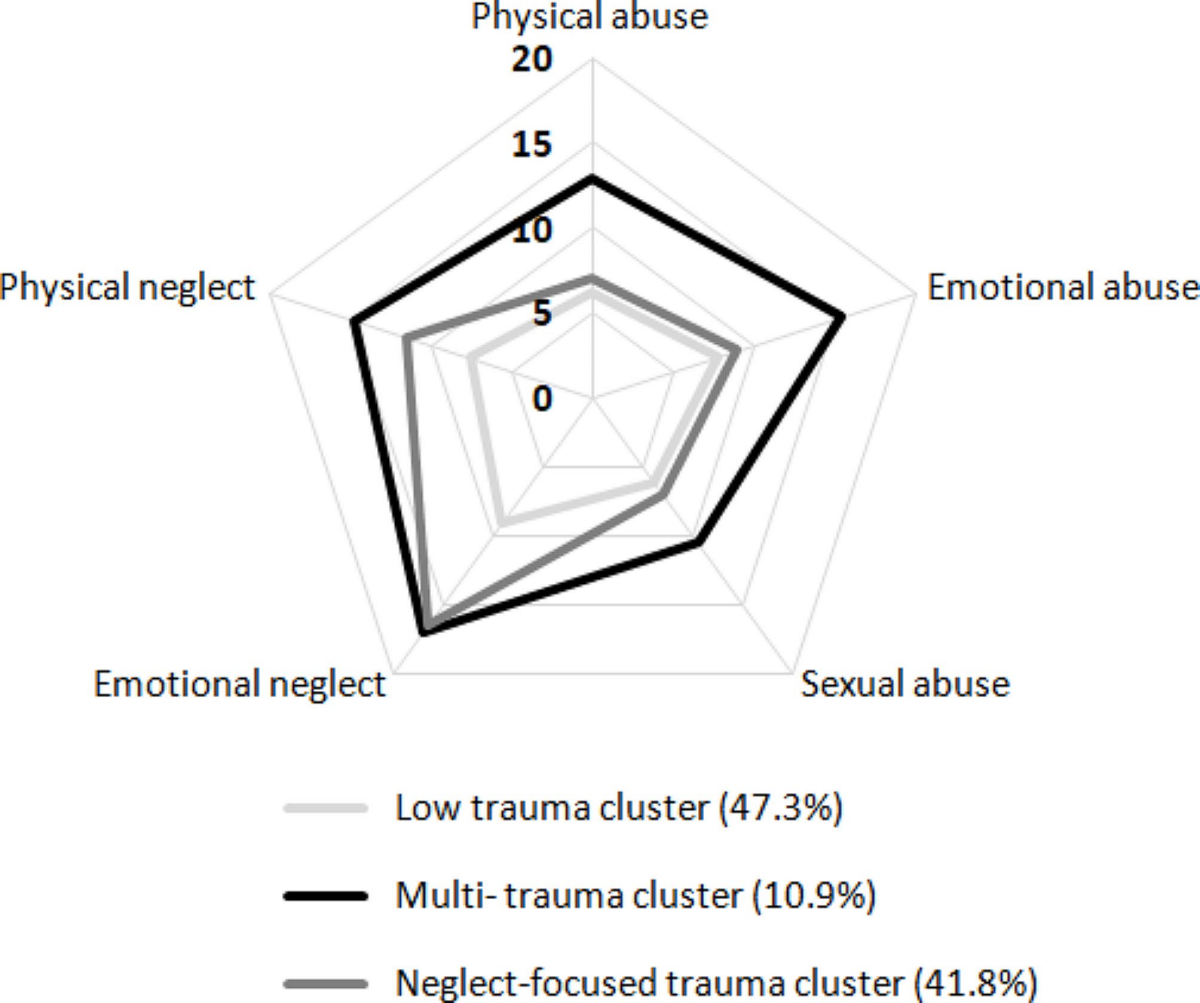
Cluster analysis of childhood trauma experience in patients with bipolar disorder

The results of cluster analysis (Fig. 1) showed one cluster with high exposure to all kinds of childhood trauma (multi-trauma cluster, 10.9%), one cluster with low exposure to all kinds of childhood trauma (low trauma cluster, 47.3%), and one cluster with especially high exposure to neglect but not abuse (neglect-focused trauma cluster, 41.8%). The distribution of clinical characteristics and cognitive profiles in different childhood trauma clusters is shown in Table 2. Gender, age, educational years, number of episodes, and other sociodemographic and clinical characteristics, including psychotropic DDD, were comparable among the three clusters. The number of childhood trauma domains was significantly different among the three clusters (*p* < 0.001). Patients in the multi-trauma cluster had the highest types of trauma exposure (average number at 4.83 ± 0.4), while the low trauma cluster and neglect-focused trauma cluster had average numbers of 1.69 ± 1.2 and 3.78 ± 0.9, respectively. There were no differences in dosage for mood stabilizers, antipsychotics, antidepressants, or benzodiazepines across the clusters. Although the prevalence of lithium use was lowest in the neglect-focused group (39.1%), it was not significantly different between clusters.

**Table 2:** Sociodemographic and clinical characteristics between childhood trauma clusters

	A. Low trauma Cluster, *N* = 26	B. Multi- trauma Cluster, *N* = 6	C. Neglect-focused trauma cluster, *N* = 23	*P*-value	Post hoc
Number of CTQ	1.69 (1.23)	4.83 (0.41)	3.78 (0.85)	0.000***	B > C > A
Physical abuse, mean (SD)	6.15 (1.75)	12.83 (5.036)	7.00 (2.08)	0.000***	B > A; B > C
Emotional abuse, mean (SD)	7.69 (2.32)	15.33 (2.42)	8.96 (2.47)	0.000***	B > A; B > C
Sexual abuse, mean (SD)	6.12 (2.30)	10.50 (3.20)	7.09 (2.15)	0.001**	B > A; B > C
Emotional neglect, mean (SD)	9.12 (2.32)	17.00 (3.63)	16.57 (3.28)	0.000***	B > A; C > A
Physical neglect, mean (SD)	7.62 (2.19)	14.67 (4.96)	11.48 (2.17)	0.000***	B > C > A
Gender, male (%)	10 (38.46)	3 (50.00)	12 (52.17)	0.612	-
Age	49.58(12.32)	45.33(13.17)	48.43(10.93)	0.728	-
Estimated premorbid IQ	103.38(9.13)	103.67(4.62)	94.27(18.25)	0.349	-
Educational years	14.00(3.74)	14.40(1.14)	12.48(2.37)	0.175	-
BMI	22.24(3.45)	22.56(2.88)	24.36(3.36)	0.089	-
Disease onset age	27.19(11.59)	28.83(9.68)	25.43(9.08)	0.708	-
Number of total episodes	8.15(4.64)	5.67(2.88)	10.09(8.60)	0.292	-
Number of manic episodes	5.38(3.72)	2.83(1.17)	6.91(7.79)	0.277	-
Number of psychotic mood episodes	2.73(2.13)	1.50(1.64)	4.87(8.48)	0.293	-
Duration of illness	22.19(10.65)	16.50(5.58)	23.45(10.38)	0.337	-
Number of suicide attempts	1.42(2.30)	0.60(0.89)	0.74(0.92)	0.329	-
Psychotropic DDD	1.86(1.319)	2.12(1.78)	2.02(1.26)	0.883	-
DDD of Mood stabilizers	0.57(0.307)	0.55(0.292)	0.54(0.472)	0.945	
Lithium, n (%)	15 (57.70)	3 (50.00)	9 (39.10)	0.431	
DDD of antipsychotics	0.79 (0.635)	0.53(0.388)	0.96(0.795)	0.367	
DDD of antidepressants	0.11 (0.317)	0.27(0.602)	0.072 (0.313)	0.451	
DDD of Benzodiazepines	0.48(0.746)	1.02(1.417)	0.51(0.549)	0.293	

CTQ, Childhood Trauma Questionnaire; IQ, Intelligence Quotient; BMI, Body Mass Index; DDD, Defined Daily Dose

*p < 0.05; ** *p* < 0.01; *** *p* < 0.001

### Cognitive profile between childhood trauma clusters

**Fig. 2:**
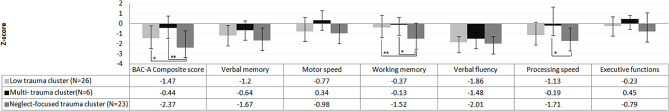
Comparison of cognitive profile between childhood trauma experience clusters. * *p* < 0.05; ** *p* < 0.01

We noted a significant difference of cognitive function between the three clusters in the BAC-A composite score, working memory, and processing speed (Fig. 2 and raw data in Supplement Table 1). Participants in the neglect-focused trauma cluster showed worse performance in the composite score and working memory compared with participants in the low trauma cluster. In addition, the neglect-focused trauma cluster had even worse performance in the composite score, working memory, and processing speed compared with participants in the multi-trauma cluster. There was no significant difference in cognitive function between participants in the multi-trauma and low trauma clusters.

### Impact of childhood trauma cluster on cognitive domains in BD patients

Generalized linear models with the low trauma cluster as a reference were used to determine the effect of the multi-trauma cluster and neglect-focused cluster on cognitive domains in BD patients (Table 3). In Model 1 adjusting for sex and age, a significant positive effect was noticed in the multi- trauma cluster in the motor speed domain compared to the low trauma cluster (ß = 1.114, *p* = 0.037). In Model 1, the neglect-focused cluster had significant negative effects in the BAC-A composite score (ß = -0.908, *p* = 0.025) and working memory (ß = -1.150, *p* = 0.002) compared to the low trauma cluster. Further, the GLM model adjusting sex, age, education year, BMI, and total psychotropic DDD (Model 2 in Table 3) consistently identified a significant negative association of the neglect-focused cluster with the BAC-A composite score (ß = -0.904, *p* = 0.025) and working memory (ß = -1.150, *p* = 0.002) compared to the low trauma cluster.

**Table 3:** Associations between childhood trauma clusters on cognitive domains in patients with bipolar disorder, reference as low trauma cluster

		Model 1		Model 2	
		ß (95% CI)	*P*-value	ß (95% CI)	*P*-value
BAC-A Composite score	Multiple trauma cluster	1.025 (-0.223–2.273)	0.108	0.778 (-0.568–2.125)	0.257
	Neglect cluster	-0.908 (-1.705–-0.118)	0.025*	-0.904 (-1.693–-0.115)	0.025*
Verbal memory	Multiple trauma cluster	0.562 (-0.406–1.531)	0.255	0.462 (-0.589–1.514)	0.389
	Neglect cluster	-0.469 (-1.082–0.143)	0.133	-0.469 (-1.086–0.147)	0.136
Motor speed	Multiple trauma cluster	1.114 (0.067–2.160)	0.037*	0.958 (-0.175–2.091)	0.097
	Neglect cluster	-0.203 (-0.865–0.458)	0.547	-0.203 (-0.867–0.461)	0.549
Working memory	Multiple trauma cluster	0.230 (-0.917–1.378)	0.694	-0.037 (-1.270–1.197)	0.954
	Neglect cluster	-1.150 (-1.875–-0.425)	0.002**	-1.150 (-1.873–-0.427)	0.002**
Verbal fluency	Multiple trauma cluster	0.382 (-0.188–0.953)	0.189	0.295 (-0.323–0.913)	0.350
	Neglect cluster	-0.149 (-0.510–0.212)	0.419	-0.149 (-0.511–0.213)	0.421
Processing speed	Multiple trauma cluster	0.940 (-0.223–2.103)	0.113	0.514 (-0.714–1.743)	0.412
	Neglect cluster	-0.581 (-1.316–0.154)	0.121	-0.581 (-1.301–0.139)	0.114
Executive functions	Multiple trauma cluster	0.677 (-0.506–1.860)	0.262	0.728 (-0.559–2.014)	0.267
	Neglect cluster	-0.554 (-1.302–0.193)	0.146	-0.554 (-1.308–0.200)	0.150

BAC-A, Brief Assessment of Cognition in Affective disorders

Model 1: Adjusted of sex, age

Model 2: Adjusted of sex, age, education year, BMI, total psychotropic DDD

* *p* < 0.05; ** *p* < 0.01; *** *p* < 0.001

## Discussion

The current study focused on a more detailed understanding of the influence of specific clusters of childhood trauma subtypes on the cognitive performance in BD patients. We found out that the multi-trauma cluster showed no difference in cognitive function compared with low trauma BD patients in the current sample. In addition, the BD patients with neglect-focused experience were associated with the worst cognitive performance among the three groups, especially in working memory. It is noteworthy that enrolled patients in this study were euthymic and with no substance use disorders. The inclusion criteria may have selected specific BD patients with “good resilience” and past multiple trauma experience. Therefore, we can further observe the negative impact of childhood neglect on cognitive function in BD patients.

Our cluster analysis results are consistent with findings of other studies investigating subjects suffering from childhood trauma experience. For example, a cross-sectional survey in Germany also revealed three childhood maltreatment clusters similar to our subgroups (Schilling et al. 2016). Adverse childhood experiences from a previous review also indicated a dose-response to unfavorable clinical outcomes in BD (Park et al. 2020). One study investigated patients with affective disorders and revealed significant cognitive impairment only in those exposed to childhood trauma compared with controls (Poletti et al. 2017). Among patients with BD and psychotic disorders, experience of abuse and neglect had higher scores on the Positive and Negative Symptoms Scale (Carbone et al. 2019).

Although it is hard to evaluate the specific impact of subtypes of childhood trauma due to the frequent co-occurrence of childhood abuse and neglect, current literature shows that both abuse and neglect are associated with cognitive performance (Mills et al. 2011). An important effect of deprivation of parental care on cognitive development in rodent and primate models has been noted, which highlights the impact of neglect on cognition (Strathearn et al. 2020). The neurodevelopmental consequences of neglect cause early problems in associative learning, which later produce problems in higher-cortical cognitive function (McLaughlin et al. 2017). There is also evidence that when taking both parental neglect and the threat of abuse into consideration, only neglect is associated with executive function (Sheridan et al. 2017). A recent BD study showed that there is a correlation between childhood trauma and global cognitive performance, while related to lower total cerebral white matter and regional abnormality over both frontal and temporal gray matter (Jørgensen et al. 2023).

Our findings suggested the BD patients under neglect childhood trauma were vulnerable to working memory deficit. The ability of working memory involves the prefrontal cortex, striatal circuits, parietal lobes, and ascending dopaminergic neuromodulatory signals (D’Esposito and Postle 2015; Higgins 2018). A meta-analysis assessing working memory showed a similar brain network with blunted activity in the striatum, anterior insula, and frontal lobe in patients with severe mental disorders, including BD (Yaple et al. 2021). More specifically, in BD, poor working memory performance has been noted with a thinner prefrontal cortex and parietal cortices (Cho and Goghari 2020), functional abnormality in the dorsolateral prefrontal cortex (DLPFC) and ventromedial prefrontal cortex (VMPFC) (Saldarini et al. 2022), and attenuated neural activation in the prefrontal cortex and posterior parietal cortex (Townsend et al. 2010). In addition, functional Val158Met polymorphism of the catechol-O-methyltransferase (*COMT*) gene, which mediates the degradation of dopamine, may also influence the aberrant activity of DLPFC during working memory performance in BD (Miskowiak et al. 2017). On the other hand, a study investigating post-institutionalized Romanian orphans showed that early deprivation with neglect could cause metabolism changes in many brain regions, including the orbital frontal gyrus, infra-limbic prefrontal cortex, medial temporal structures, lateral temporal cortex, and brain stem (Chugani et al. 2001). A study specifically focused on BD also showed that both physical and emotional neglect are associated with a dysregulated frontoparietal circuit, and physical neglect may specifically impact the functional connectivity of the left-caudate-seed to the frontoparietal network (Hsieh et al. 2021). Therefore, we notified that the brain network involved in subjects with childhood neglect corresponds to the brain area for working memory in BD. Furthermore, research has suggested that in a deprived early environment, there may be excessive synaptic pruning and problems with myelination, causing reductions in cortical thickness and white matter integrity (McLaughlin et al. 2017). In addition, other mechanisms also link early life stress to cognitive outcomes, including interaction with genotypes, epigenetic modification, behavioral adaptation, and the impact on the HPA axis, immune system, oxidative stress, and alteration in neurotrophin factors (Aas et al. 2019; Deighton et al. 2018; Grillault Laroche et al. 2020; Horn et al. 2019; Jaworska-Andryszewska and Rybakowski 2019; Jiang et al. 2019; Maes et al. 2022; Tyrka et al. 2013).

It is interesting to note that in this study, the multi-trauma cluster unexpectedly did not show the worst cognitive performance. These findings indicated that the impact of adverse childhood experience on cognition does not simply rely on the cumulative effect of different childhood traumas, and some cluster characteristics need to be mentioned. For example, in the current study, we excluded BD patients with substance use disorder, and the cluster with multi-trauma showed trend of older onset age, fewer total episodes, and shorter duration of the illness. Those who could remain stable could be a special subpopulation in this multi-trauma cluster, which may be protected from some other biological or psychological factors. For example, they could have larger gray and whiter matter volume in the hippocampus and greater connectivity between the central executive network and the limbic regions, as shown in the population with resilience remaining after exposure to childhood maltreatment (Moreno-López et al. 2020).

With a clustering method instead of a categorical method, the current study provides further understanding of common distributions of childhood trauma experience in BD and the unique profile of their cognitive performance. The results allowed us to evaluate the impact of a certain combination of childhood trauma subtypes and provided a more realistic point of view. But there are several limitations that should be considered in this study. First, childhood trauma experience was assessed via self-report, which inevitably faces the issue of recall bias and is often questioned for the possible influence of patients’ psychopathology. However, CTQ scales have proven validity and reliability in both psychotic patients (Fisher et al. 2011) and a BD population (Hosang et al. 2023). Second, more detailed information is lacking about the childhood trauma, such as the actual frequency and duration, which may lead to an over-simplified model for the understanding of trauma experience. Third, all of the participants were recruited from a single tertiary psychiatric hospital. Therefore, the patients likely had a more severe degree of illness and cognitive dysfunction, causing uncertainty about generalizability of the study. Fourth, the sample size in our study was small, leading to limited sizes for each cluster. Only 10% of the patients clustered into the multi-trauma group, making it difficult to demonstrate differences in performance of cognitive domains due to small sample size. A larger sample size of multi-trauma cluster might reveal a cognitive profile different from both low trauma cluster and neglect-focused trauma cluster. Fifth, while assessing cognitive performances, it’s important to consider the impact of medications. However, the effects of different categories of pharmacological treatment on cognitive deficits in BD have shown mixed results in previous studies (Sanches et al. 2015; Wingo et al. 2009; Xu et al. 2020; Yatham et al. 2017). Although there was no significant difference in psychotropic DDD between clusters, we noted a relatively low proportion of subjects receiving lithium treatment in the neglect-focus group, while the long-term beneficial effect of lithium to BD on their cognition compared with other anticonvulsants still needed more research for precise conclusions (Sabater et al. 2016). Lastly, we did not have longitudinal cognitive profiles to ensure the cognitive stability of the patients.

In conclusion, this study confirmed that neglect-focused trauma experience in BD may cause a negative impact on working memory function. There might be a unique influence of the neglect cluster over childhood abuse. Findings from this study suggest that in clinical practice, history of childhood trauma experiences, not only abuse but also neglect, should be assessed in BD population as it may be associated with cognitive deficits and require further attention while managing the patients. Further research is warranted for understanding the linking mechanisms of childhood neglect, such as disturbed neurodevelopmental process, neurocircuits, immune and inflammatory system. Specific interventions focusing on preventing cognitive deterioration are also required in this cluster of patients.

### Electronic supplementary material

Below is the link to the electronic supplementary material.

Supplementary Material 1

## Data Availability

The data that support the findings of this study are available on request from the corresponding author.
