# Supplementary material for: Cluster analysis exploring the impact of childhood neglect on cognitive function in patients with bipolar disorder
Source: Int J Bipolar Disord. 2024 Apr 27;12:13. doi: 10.1186/s40345-024-00335-w (PMC11055839; doi:10.1186/s40345-024-00335-w)
Supplement: Supplementary file 1 — Supplementary Material 1 [file 40345_2024_335_MOESM1_ESM.docx]

Supplement Table 1. Comparison of cognitive profile between three clusters.

|  | 1. Low trauma cluster  (N=26) | 1. Multi- trauma cluster   (N=6) | 1. Neglect-focused trauma cluster  (N=23) | P-value | Post hoc |
| --- | --- | --- | --- | --- | --- |
| BAC-A Composite score | -1.47 (1.26) | -0.44 (1.22) | -2.37 (1.67) | 0.010* | A>C; B>C |
| Verbal memory | -1.20 (1.03) | -0.64 (0.92) | -1.67 (1.26) | 0.104 | - |
| Motor speed | -0.77 (1.40) | 0.34 (0.97) | -0.98 (1.01) | 0.068 | - |
| Working memory | -0.37 (1.18) | -0.13 (0.75) | -1.52 (1.57) | 0.007** | A>C; B>C |
| Verbal fluency | -1.86 (0.59) | -1.48 (0.61) | -2.01 (0.75) | 0.220 | - |
| Processing speed | -1.13 (1.27) | -0.19 (1.88) | -1.71 (1.29) | 0.045* | B>C |
| Executive functions | -0.23 (0.91) | 0.45 (0.39) | -0.79 (1.86) | 0.116 | - |

BAC-A, Brief Assessment of Cognition in Affective disorders

**p* < 0.05; ** *p* < 0.01
